# Poly[diacetonitrile­[μ_3_-difluoro­(oxalato)borato]sodium]

**DOI:** 10.1107/S1600536811015091

**Published:** 2011-05-07

**Authors:** Joshua L. Allen, Paul D. Boyle, Wesley A. Henderson

**Affiliations:** a Ionic Liquids and Electrolytes for Energy Technologies (ILEET) Laboratory, Department of Chemical and Biomolecular Engineering, North Carolina State University, 911 Partners Way, Raleigh, NC 27695, USA; b X-ray Structural Facility, Department of Chemistry, North Carolina State University, 2620 Yarbrough Drive, Raleigh, NC 27695, USA

## Abstract

The title compound, [Na(C_2_BF_2_O_4_)(CH_3_CN)_2_]_*n*_, forms infinite two-dimensional layers running parallel to (010). The layers lie across crystallographic mirror planes at *y* = 1/4 and 3/4. The Na, B and two F atoms reside on these mirror planes. The Na^+^ cations are six-coordinate. Two equatorial coordination positions are occupied by acetonitrile mol­ecules. The other two equatorial coordination sites are occupied by the chelating O atoms from the difluoro­(oxalato)borate anion (DFOB^−^). The axial coordination sites are occupied by two F atoms from two different DFOB^−^ anions.

## Related literature

For the electrochemical properties of the DFOB^−^ anion, see: Zhang (2007 ▶); Chen *et al.* (2007 ▶); Fu *et al.* (2010 ▶). For ionic liquids based on the DFOB^−^ anion, see: Schreiner *et al.* (2009 ▶). For the benefits of ionic liquid additives in Li^+^ ion batteries, see: Kim *et al.* (2010 ▶); Schreiner *et al.* (2009 ▶); Sugimoto *et al.* (2009 ▶); Moosbauer *et al.* (2010 ▶). 
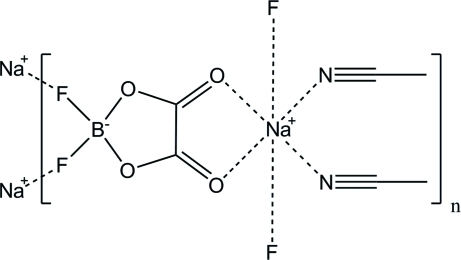


## Experimental

### 

#### Crystal data

[Na(C_2_BF_2_O_4_)(C_2_H_3_N)_2_]
*M* *_r_* = 241.93
Orthorhombic, 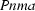

*a* = 11.6932 (3) Å
*b* = 14.1254 (3) Å
*c* = 6.5130 (1) Å
*V* = 1075.76 (4) Å^3^
*Z* = 4
Mo *K*α radiation
μ = 0.17 mm^−1^
*T* = 110 K
0.38 × 0.37 × 0.23 mm

#### Data collection

Bruker–Nonius Kappa-axis X8 APEXII diffractometer
Absorption correction: multi-scan (*SADABS*; Bruker, 2009 ▶) *T*_min_ = 0.938, *T*_max_ = 0.961
33523 measured reflections
3243 independent reflections
2599 reflections with *I* > 2σ(*I*)
*R*_int_ = 0.032

#### Refinement

*R*[*F* ^2^ > 2σ(*F* ^2^)] = 0.037
*wR*(*F* ^2^) = 0.112
*S* = 1.09
3243 reflections
81 parameters
H-atom parameters constrained
Δρ_max_ = 0.47 e Å^−3^
Δρ_min_ = −0.33 e Å^−3^

### 

Data collection: *APEX2* (Bruker, 2009 ▶); cell refinement: *SAINT* (Bruker, 2009 ▶); data reduction: *SAINT*; program(s) used to solve structure: *SHELXS* (Sheldrick, 2008 ▶); program(s) used to refine structure: *SHELXTL* (Sheldrick, 2008 ▶); molecular graphics: *ORTEP-3* (Farrugia, 1997 ▶); software used to prepare material for publication: *cif2tables.py* (Boyle, 2008 ▶).

## Supplementary Material

Crystal structure: contains datablocks I, global. DOI: 10.1107/S1600536811015091/sj5126sup1.cif

Structure factors: contains datablocks I. DOI: 10.1107/S1600536811015091/sj5126Isup2.hkl

Additional supplementary materials:  crystallographic information; 3D view; checkCIF report

## Figures and Tables

**Fig. 1.**
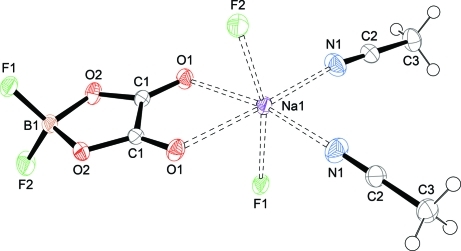
Asymmetric unit of (AN)2:NaDFOB reflected across its mirror plane with naming/numbering scheme. Thermal ellipsoids are at 50% probability (Na-purple, O-red, F-green, B-tan, C-grey, N-blue).

**Fig. 2.**
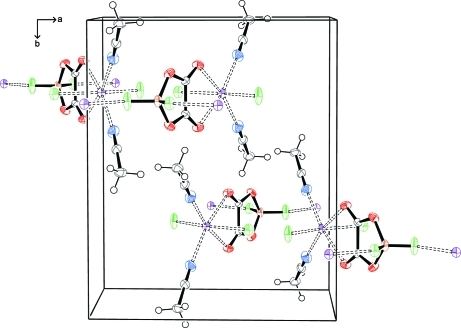
Ion and solvent coordination in (AN)2:NaDFOB. Thermal ellipsoids are at 50% probability (Na-purple, O-red, F-green, B-tan, C-grey, N-blue).

**Fig. 3.**
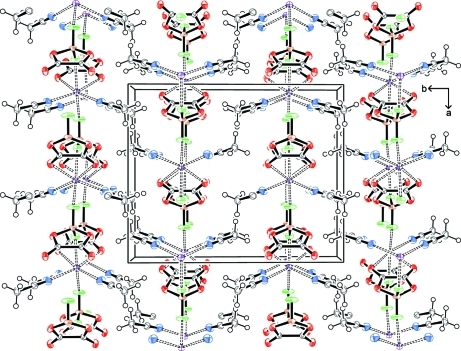
Packing diagram of (AN)2:NaDFOB. Thermal ellipsoids are at 50% probability (Na-purple, O-red, F-green, B-tan, C-grey, N-blue).

## supplementary crystallographic information

### Comment

Ionic liquids (ILs) have attracted much attention recently, especially room-temperature ionic liquids (RTILs), due to their many favorable properties including large liquid range, high conductivity, low vapor pressure, tailorable hydrophobicity and high thermal stability. Recently, a new class of ILs based upon the DFOB^-^ anion has been reported (Schreiner *et al.*, 2009). These ILs may contribute extensively to the solid-electrolyte interface (SEI) formation in Li-ion batteries (Zhang, 2007; Chen *et al.*, 2007; Fu *et al.*, 2010), especially when used as an additive (Kim *et al.*, 2010; Sugimoto *et al.*, 2009; Moosbauer *et al.*, 2010). The synthesis of ILs based on the DFOB^-^ anion has thus far been a multi-step process involving the synthesis of tetrafluoroborate BF_4_^-^-based ILs, which are then reacted to displace two F atoms with an oxalate moiety to form DFOB^-^ anions. The title compound, sodium difluoro(oxalato)borate (NaDFOB), can be used as an alternative reagent for the synthesis of DFOB^-^-based ILs by reacting it directly with bromide salts with organic cations producing the DFOB^-^-based ILs and NaBr. Therefore, the title compound may become an important reagent for use in the synthesis of ILs for Li-ion battery electrolytes.

The Na^+^ cation in the title structure, which resides on a crystallographic mirror plane, is coordinated by two carbonyl O atoms from a single DFOB^-^ anion, two F atoms from two distinct DFOB^-^ anions and two N atoms from two acetonitrile molecules (Fig. 1). The pseudo-octahedral structure is packed in the crystal structure such that *Z* = 4 (Fig. 2), forming two dimensional layers in which acetonitrile molecules form the exterior of the layer (Fig. 3). The shortest C···C contact between the acetonitrile exteriors of the layers is 3.675 Å.

### Experimental

NaDFOB was synthesized by the direct reaction of excess boron trifluoride diethyl etherate (BF_3_-ether) with sodium oxalate, both used as-received from Sigma-Aldrich. The resulting salt was extracted with anhydrous acetonitrile (Sigma-Aldrich) by dissolving the NaDFOB and filtering off the NaF solid byproduct. NaDFOB was allowed to slow crystallize at -20°C forming colorless crystals suitable for X-ray analysis.

### Refinement

The structure was solved by direct methods using the *XS* program. All non-hydrogen atoms were obtained from the initial solution. The hydrogen atoms were introduced at idealized positions and were allowed to ride on the parent carbon atom. The CH_3_ orientation and the C—H distance were allowed to vary during the refinement. The structural model was fit to the data using full matrix least-squares based on *F*^2^. The calculated structure factors included corrections for anomalous dispersion from the usual tabulation. The structure was refined using the *XL* program from *SHELXTL*, graphic plots were produced using the *ORTEP-3* crystallographic program suite.

### Crystal data

**Table:**

[Na(C_2_BF_2_O_4_)(C_2_H_3_N)_2_]	*F*(000) = 488
*M**_r_* = 241.93	*D*_x_ = 1.494 Mg m^−^^3^
Orthorhombic, *P**n**m**a*	Mo *K*α radiation, λ = 0.71073 Å
Hall symbol: -P 2ac 2n	Cell parameters from 9940 reflections
*a* = 11.6932 (3) Å	θ = 3.4–38.5°
*b* = 14.1254 (3) Å	µ = 0.17 mm^−^^1^
*c* = 6.5130 (1) Å	*T* = 110 K
*V* = 1075.76 (4) Å^3^	Prism, colorless
*Z* = 4	0.38 × 0.37 × 0.23 mm

### Data collection

**Table:**

Bruker–Nonius Kappa-axis X8 APEXII diffractometer	3243 independent reflections
Radiation source: fine-focus sealed tube	2599 reflections with *I* > 2σ(*I*)
graphite	*R*_int_ = 0.032
ω and φ scans	θ_max_ = 39.4°, θ_min_ = 3.4°
Absorption correction: multi-scan (*SADABS*; Bruker, 2009)	*h* = −20→20
*T*_min_ = 0.938, *T*_max_ = 0.961	*k* = −25→22
33523 measured reflections	*l* = −11→11

### Refinement

**Table:**

Refinement on *F*^2^	Primary atom site location: structure-invariant direct methods
Least-squares matrix: full	Secondary atom site location: difference Fourier map
*R*[*F*^2^ > 2σ(*F*^2^)] = 0.037	Hydrogen site location: inferred from neighbouring sites
*wR*(*F*^2^) = 0.112	H-atom parameters constrained
*S* = 1.09	*w* = 1/[σ^2^(*F*_o_^2^) + (0.0573*P*)^2^ + 0.1343*P*] where *P* = (*F*_o_^2^ + 2*F*_c_^2^)/3
3243 reflections	(Δ/σ)_max_ = 0.001
81 parameters	Δρ_max_ = 0.47 e Å^−^^3^
0 restraints	Δρ_min_ = −0.33 e Å^−^^3^

### Special details

**Table:**

Geometry. All e.s.d.'s (except the e.s.d. in the dihedral angle between two l.s. planes) are estimated using the full covariance matrix. The cell e.s.d.'s are taken into account individually in the estimation of e.s.d.'s in distances, angles and torsion angles; correlations between e.s.d.'s in cell parameters are only used when they are defined by crystal symmetry. An approximate (isotropic) treatment of cell e.s.d.'s is used for estimating e.s.d.'s involving l.s. planes.
Refinement. Refinement of *F*^2^ against ALL reflections. The weighted *R*-factor *wR* and goodness of fit *S* are based on *F*^2^, conventional *R*-factors *R* are based on *F*, with *F* set to zero for negative *F*^2^. The threshold expression of *F*^2^ > σ(*F*^2^) is used only for calculating *R*-factors(gt) *etc*. and is not relevant to the choice of reflections for refinement. *R*-factors based on *F*^2^ are statistically about twice as large as those based on *F*, and *R*- factors based on ALL data will be even larger.

### Fractional atomic coordinates and isotropic or equivalent isotropic displacement parameters (Å^2^)

**Table:**

	*x*	*y*	*z*	*U*_iso_*/*U*_eq_	
Na1	0.53868 (3)	0.2500	0.20509 (6)	0.01770 (9)	
O1	0.44700 (5)	0.14747 (4)	−0.05163 (8)	0.02226 (11)	
O2	0.34387 (5)	0.16659 (4)	−0.34157 (8)	0.02147 (11)	
C1	0.40112 (5)	0.19563 (4)	−0.18020 (9)	0.01607 (11)	
B1	0.30460 (9)	0.2500	−0.46233 (16)	0.01987 (18)	
F1	0.35481 (6)	0.2500	−0.65402 (9)	0.02390 (13)	
F2	0.18741 (6)	0.2500	−0.47948 (11)	0.0361 (2)	
N1	0.60143 (7)	0.12710 (6)	0.43675 (12)	0.03178 (16)	
C2	0.62179 (6)	0.07466 (5)	0.56558 (11)	0.02313 (13)	
C3	0.64674 (7)	0.00803 (5)	0.72867 (13)	0.02731 (15)	
H3A	0.6132 (7)	0.0290 (3)	0.8509 (11)	0.041*	
H3B	0.7260 (7)	0.0035 (4)	0.7461 (9)	0.041*	
H3C	0.6171 (7)	−0.0515 (5)	0.6943 (7)	0.041*	

### Atomic displacement parameters (Å^2^)

**Table:**

	*U*^11^	*U*^22^	*U*^33^	*U*^12^	*U*^13^	*U*^23^
Na1	0.01740 (17)	0.02188 (18)	0.01382 (16)	0.000	−0.00027 (12)	0.000
O1	0.0307 (3)	0.0182 (2)	0.0179 (2)	0.00205 (17)	−0.00250 (18)	0.00271 (15)
O2	0.0256 (2)	0.0215 (2)	0.0174 (2)	−0.00691 (17)	−0.00341 (17)	−0.00137 (16)
C1	0.0167 (2)	0.0164 (2)	0.0151 (2)	−0.00148 (17)	0.00062 (18)	−0.00046 (17)
B1	0.0140 (4)	0.0303 (5)	0.0153 (4)	0.000	−0.0009 (3)	0.000
F1	0.0217 (3)	0.0354 (3)	0.0146 (2)	0.000	0.0017 (2)	0.000
F2	0.0139 (3)	0.0714 (6)	0.0230 (3)	0.000	−0.0036 (2)	0.000
N1	0.0300 (3)	0.0352 (3)	0.0301 (3)	0.0053 (3)	−0.0019 (3)	0.0104 (3)
C2	0.0211 (3)	0.0235 (3)	0.0247 (3)	0.0018 (2)	−0.0018 (2)	0.0026 (2)
C3	0.0334 (4)	0.0199 (3)	0.0287 (3)	−0.0007 (2)	−0.0077 (3)	0.0053 (2)

### Geometric parameters (Å, °)

**Table:**

Na1—N1	2.4143 (8)	C1—C1^i^	1.5360 (12)
Na1—N1^i^	2.4143 (8)	B1—F2	1.3748 (12)
Na1—F2^ii^	2.2768 (8)	B1—F1	1.3797 (12)
Na1—F1^iii^	2.3377 (7)	B1—O2^i^	1.4892 (8)
Na1—O1	2.4582 (6)	F1—Na1^iv^	2.3377 (7)
Na1—O1^i^	2.4582 (6)	F2—Na1^v^	2.2768 (8)
Na1—C1	3.0780 (7)	N1—C2	1.1443 (10)
Na1—C1^i^	3.0780 (7)	C2—C3	1.4489 (10)
O1—C1	1.2049 (8)	C3—H3A	0.9359
O2—C1	1.3119 (8)	C3—H3B	0.9359
O2—B1	1.4892 (8)	C3—H3C	0.9359
			
F2^ii^—Na1—F1^iii^	162.92 (3)	O1^i^—Na1—C1^i^	21.651 (16)
F2^ii^—Na1—N1^i^	99.86 (3)	C1—Na1—C1^i^	28.90 (2)
F1^iii^—Na1—N1^i^	91.96 (2)	C1—O1—Na1	109.52 (4)
F2^ii^—Na1—N1	99.86 (3)	C1—O2—B1	109.46 (5)
F1^iii^—Na1—N1	91.96 (2)	O1—C1—O2	127.40 (6)
N1^i^—Na1—N1	91.96 (4)	O1—C1—C1^i^	124.38 (4)
F2^ii^—Na1—O1	83.93 (2)	O2—C1—C1^i^	108.22 (4)
F1^iii^—Na1—O1	82.29 (2)	O1—C1—Na1	48.83 (3)
N1^i^—Na1—O1	168.90 (3)	O2—C1—Na1	176.22 (4)
N1—Na1—O1	97.70 (2)	C1^i^—C1—Na1	75.551 (11)
F2^ii^—Na1—O1^i^	83.93 (2)	F2—B1—F1	110.52 (8)
F1^iii^—Na1—O1^i^	82.29 (2)	F2—B1—O2^i^	110.50 (5)
N1^i^—Na1—O1^i^	97.70 (2)	F1—B1—O2^i^	110.29 (5)
N1—Na1—O1^i^	168.90 (3)	F2—B1—O2	110.50 (5)
O1—Na1—O1^i^	72.20 (3)	F1—B1—O2	110.29 (5)
F2^ii^—Na1—C1	82.71 (2)	O2^i^—B1—O2	104.59 (7)
F1^iii^—Na1—C1	80.76 (2)	B1—F1—Na1^iv^	138.30 (6)
N1^i^—Na1—C1	147.97 (2)	B1—F2—Na1^v^	144.47 (6)
N1—Na1—C1	119.27 (2)	C2—N1—Na1	170.74 (7)
O1—Na1—C1	21.651 (16)	N1—C2—C3	179.60 (9)
O1^i^—Na1—C1	50.549 (18)	C2—C3—H3A	109.5
F2^ii^—Na1—C1^i^	82.71 (2)	C2—C3—H3B	109.5
F1^iii^—Na1—C1^i^	80.76 (2)	H3A—C3—H3B	109.5
N1^i^—Na1—C1^i^	119.27 (2)	C2—C3—H3C	109.5
N1—Na1—C1^i^	147.97 (2)	H3A—C3—H3C	109.5
O1—Na1—C1^i^	50.549 (18)	H3B—C3—H3C	109.5
			
F2^ii^—Na1—O1—C1	−85.53 (5)	F2^ii^—Na1—C1—C1^i^	−88.110 (6)
F1^iii^—Na1—O1—C1	84.36 (5)	F1^iii^—Na1—C1—C1^i^	87.597 (6)
N1^i^—Na1—O1—C1	25.08 (17)	N1^i^—Na1—C1—C1^i^	8.83 (5)
N1—Na1—O1—C1	175.30 (5)	N1—Na1—C1—C1^i^	174.65 (3)
O1^i^—Na1—O1—C1	0.02 (5)	O1—Na1—C1—C1^i^	179.98 (5)
C1^i^—Na1—O1—C1	0.01 (3)	O1^i^—Na1—C1—C1^i^	0.01 (2)
Na1—O1—C1—O2	179.80 (5)	C1—O2—B1—F2	−121.16 (7)
Na1—O1—C1—C1^i^	−0.02 (5)	C1—O2—B1—F1	116.34 (6)
B1—O2—C1—O1	−178.41 (7)	C1—O2—B1—O2^i^	−2.23 (9)
B1—O2—C1—C1^i^	1.44 (6)	F2—B1—F1—Na1^iv^	180.0
B1—O2—C1—Na1	−176.1 (7)	O2^i^—B1—F1—Na1^iv^	57.51 (5)
F2^ii^—Na1—C1—O1	91.91 (5)	O2—B1—F1—Na1^iv^	−57.51 (5)
F1^iii^—Na1—C1—O1	−92.38 (5)	F1—B1—F2—Na1^v^	0.0
N1^i^—Na1—C1—O1	−171.15 (6)	O2^i^—B1—F2—Na1^v^	122.36 (5)
N1—Na1—C1—O1	−5.34 (6)	O2—B1—F2—Na1^v^	−122.36 (5)
O1^i^—Na1—C1—O1	−179.97 (7)	F2^ii^—Na1—N1—C2	156.5 (5)
C1^i^—Na1—C1—O1	−179.98 (5)	F1^iii^—Na1—N1—C2	−35.9 (5)
F2^ii^—Na1—C1—O2	89.5 (7)	N1^i^—Na1—N1—C2	56.1 (5)
F1^iii^—Na1—C1—O2	−94.8 (7)	O1—Na1—N1—C2	−118.4 (5)
N1^i^—Na1—C1—O2	−173.6 (7)	O1^i^—Na1—N1—C2	−94.4 (5)
N1—Na1—C1—O2	−7.7 (7)	C1—Na1—N1—C2	−116.4 (5)
O1—Na1—C1—O2	−2.4 (7)	C1^i^—Na1—N1—C2	−111.6 (5)
O1^i^—Na1—C1—O2	177.6 (7)	Na1—N1—C2—C3	66 (14)
C1^i^—Na1—C1—O2	177.6 (7)		

Symmetry codes: (i) *x*, −*y*+1/2, *z*; (ii) *x*+1/2, −*y*+1/2, −*z*−1/2; (iii) *x*, *y*, *z*+1; (iv) *x*, *y*, *z*−1; (v) *x*−1/2, −*y*+1/2, −*z*−1/2.
